# Supplementary material for: Dual-Drug Loaded Separable Microneedles for Efficient Rheumatoid Arthritis Therapy
Source: Pharmaceutics. 2022 Jul 21;14(7):1518. doi: 10.3390/pharmaceutics14071518 (PMC9324764; doi:10.3390/pharmaceutics14071518)
Supplement: Supplementary file 1 [file pharmaceutics-14-01518-s001.zip › pharmaceutics-1769554-supplementary.pdf]

# Dual-drug loaded separable microneedles for efficient rheumatoid arthritis therapy

Mengchen An[a,b,c], Mengxiao Shi [a,b,c], Jingjing Su [a,b,c], Yueru Wei [a,b,c], Rongrong Luo[a,b,c], Pengchao Sun[a,b,c,*], Yongxing Zhao[a,b,c,*]

[a]Department of Pharmaceutics, School of Pharmaceutical Sciences, Zhengzhou University, No.100 Science Ave, Zhengzhou 450001, PR China

[b]Key Laboratory of Targeting Therapy and Diagnosis for Critical Diseases, Zhengzhou University, No.100 Science Ave, Zhengzhou 450001, PR China

[c]Key Laboratory of Advanced Pharmaceutical Technology, Ministry of Education of China, Zhengzhou University, Zhengzhou 450001, PR China

[*]These are corresponding authors. Address: Department of Pharmaceutics, School of Pharmaceutical Sciences, Zhengzhou University, No.100 Kexue Ave, Zhengzhou 450001, PR China; Emails: pengchao_sun@zzu.edu.cn, zhaoyx@zzu.edu.cn

# Supplementary materials

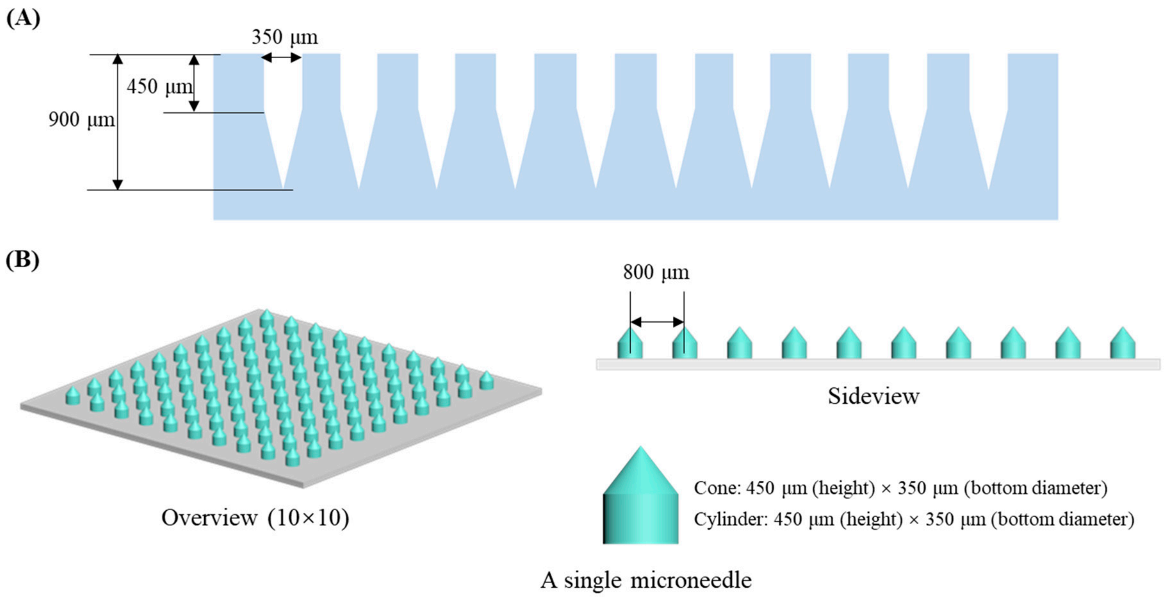


**Figure S1** Overview of the designed MN. (A) Side view of the PDMS mold. (B) Master mold.

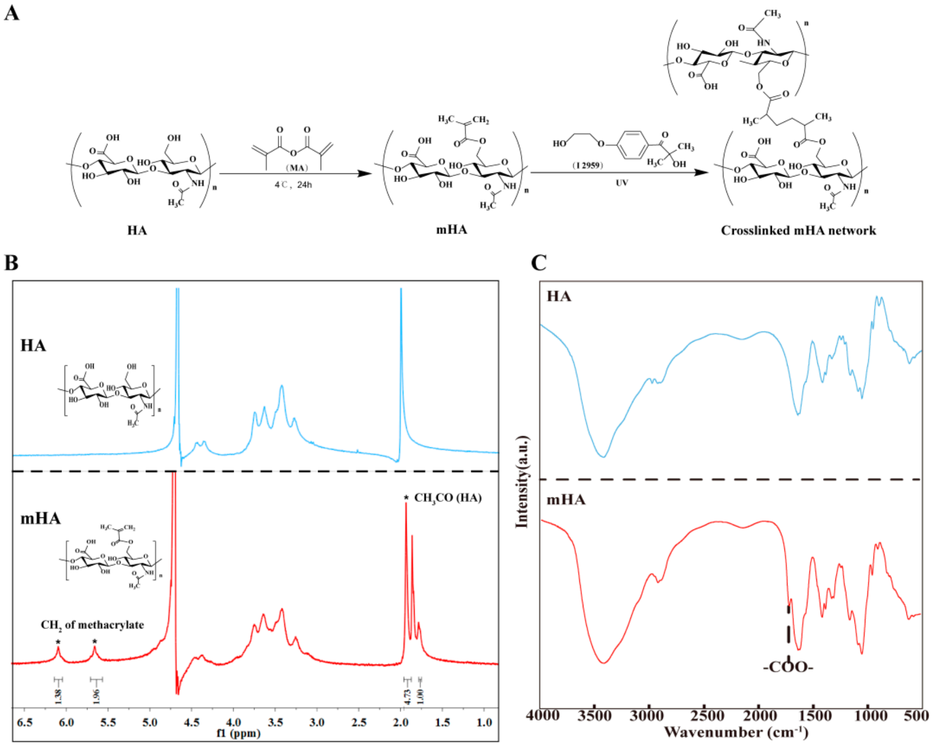


**Figure S2** Synthesis and characterization of methacrylate modified hyaluronic acid (mHA). (A) Synthesis of mHA and UV initiated crosslinking. (B) $^{1}$H-NMR spectra of HA and mHA.

(C) FTIR spectra of HA and mHA.

HA gained carbon-carbon double bonds after esterification with methacrylate anhydride, which leads to the cross-linking of the network after UV irradiation. The characteristic peaks at ~6.1 ppm and ~5.7 ppm represented the methacrylate protons, while the peak at ~1.9 ppm represented methyl proton signals of HA, indicating the successful preparation of mHA (Figure S2B). The successful synthesis of mHA was further verified by FTIR with a characteristic absorption at ~1700 $cm^{-1}$ (Figure S2C).

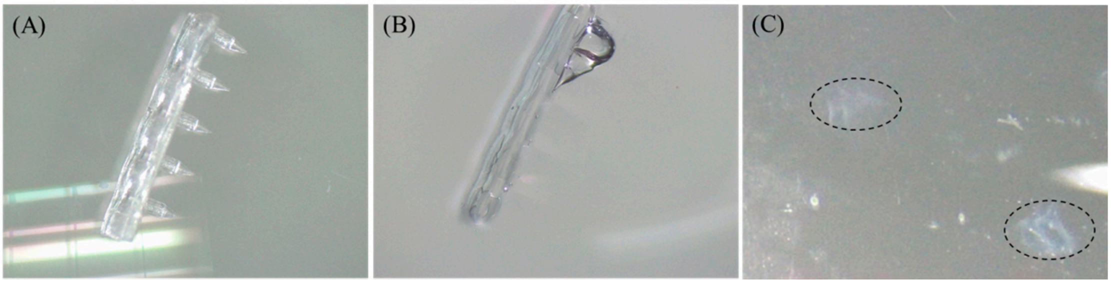


**Figure S3** The separation of MN. (A) Before water rinse. (B) Dissolution of the needle body. (C) Separated needle tips.

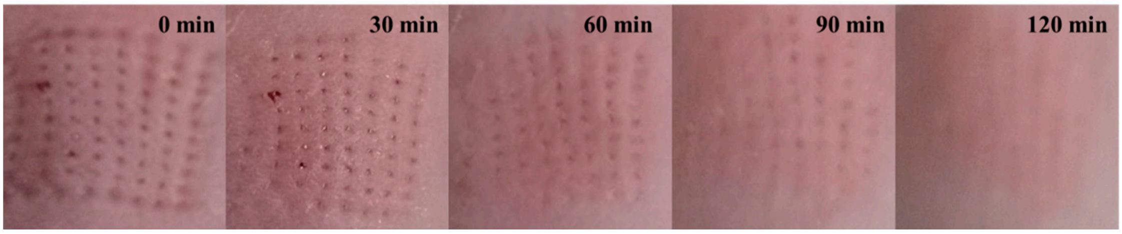


**Figure S4** Skin recovery after application of mHA prepared MN.

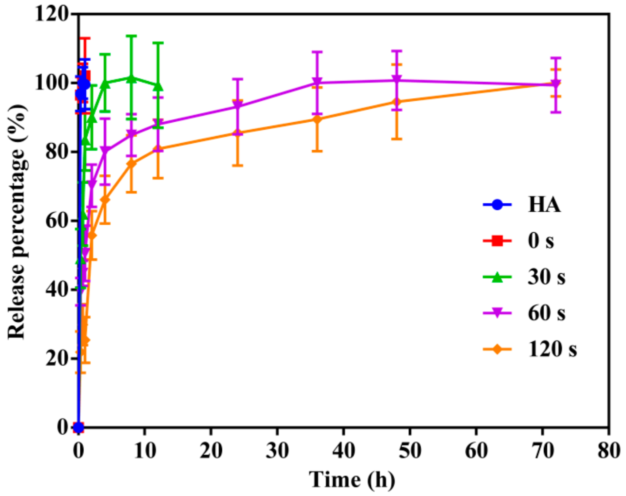


**Figure S5** *In vitro* release of BSA-FITC from the needle tips of the separable MN (0~80 h).

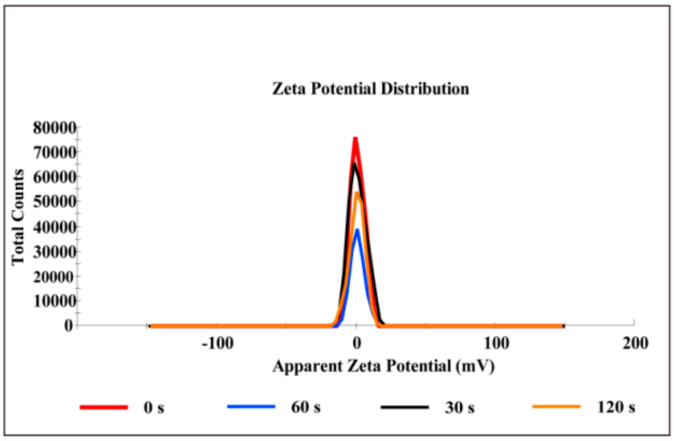


**Figure S6** Zeta potential of TCZ after UV irradiation.

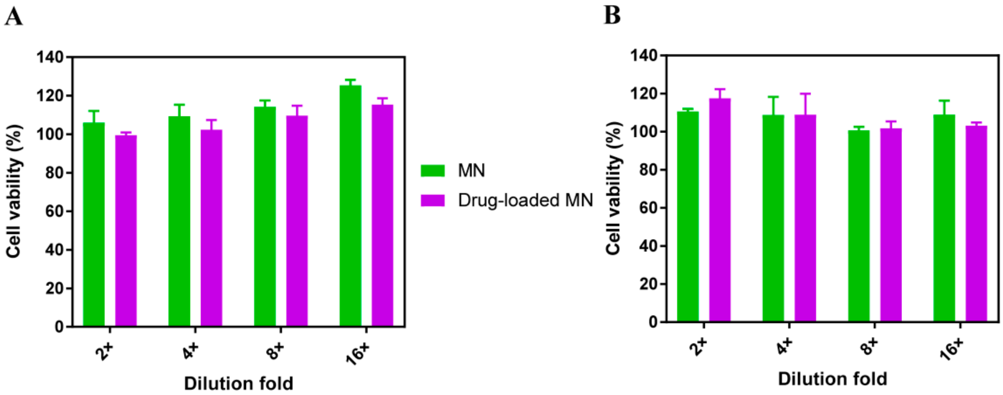


**Figure S7** The cytotoxicity of MN and drug-loaded MN against murine fibroblasts L929 (A) and human fibroblasts FLS (B).

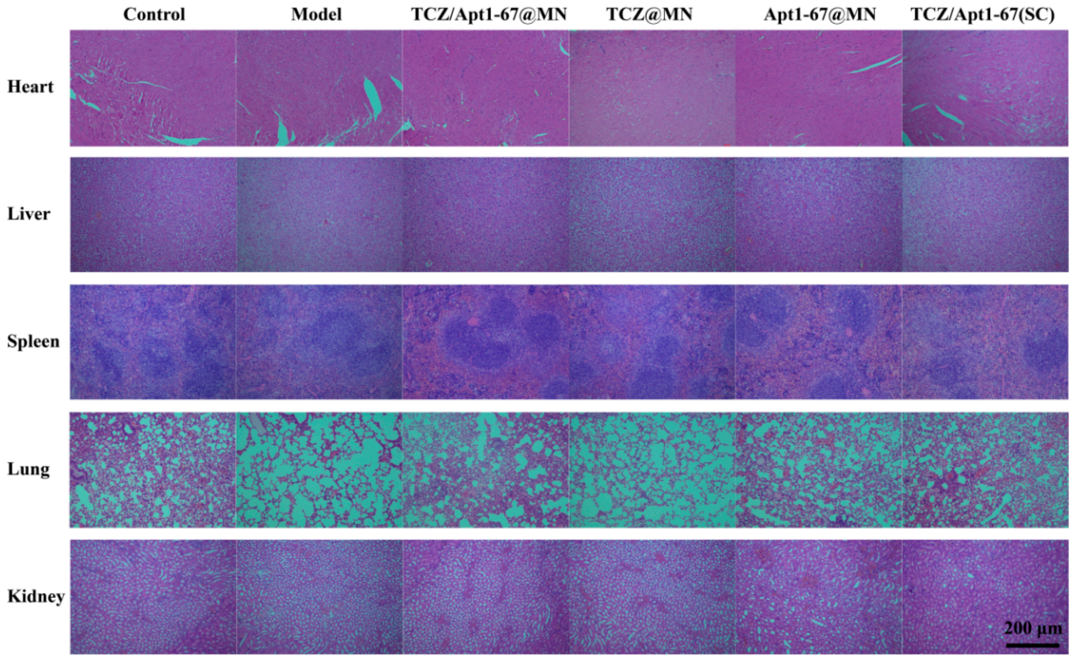


**Figure S8** Influences of various treatments on the organs of mice.

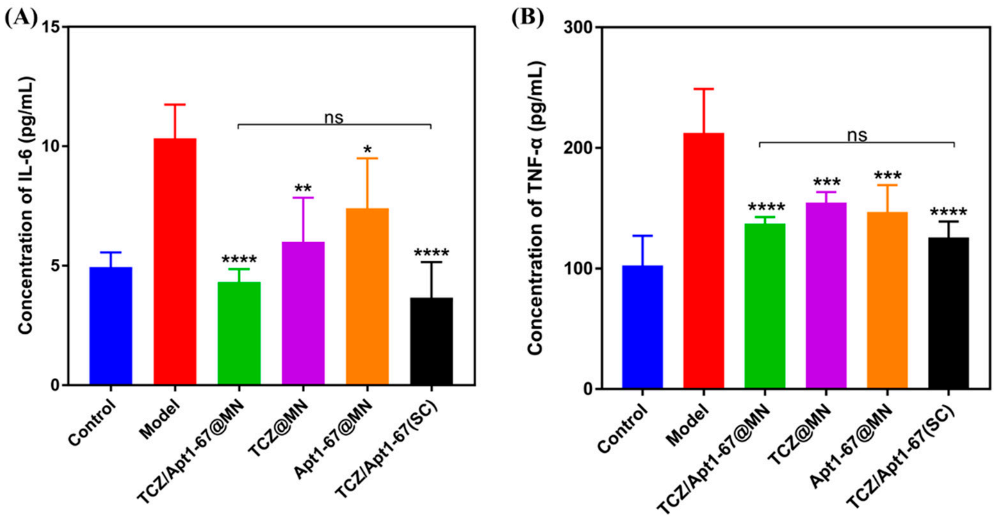


**Figure S9** Serum levels of IL6 (A) and TNF-$\alpha$ (B). *p＜0.05,**p＜0.01, ***p＜0.001，****p＜0.0001, ns: not significant
